# How emotions shape memory? The moderating effect of healthy emotionality on eyewitness testimony

**DOI:** 10.3389/fpsyg.2024.1406897

**Published:** 2024-06-06

**Authors:** Kaja Glomb, Przemysław Piotrowski, Bożena Gulla, Iza Romanowska, Maria Mastek

**Affiliations:** ^1^Faculty of Management and Social Communication, Jagiellonian University in Krakow, Krakow, Poland; ^2^Center for Humanities Computing (CHC), University of Aarhus, Aarhus, Denmark

**Keywords:** memory, emotionality, individual differences, face identification, free recall

## Abstract

The influence of emotions on memory is a significant topic in the psychology of eyewitness testimony. However, conflicting results have arisen, possibly due to varying approaches and methodologies across studies. These discrepancies might also arise from inadequate consideration of individual differences in emotionality. Therefore, this study aims to analyze the moderating effect of healthy emotionality on the relationship between emotion and memory of criminal events. The results of our laboratory experiment (*N* = 150) conducted with VR technology indicate that eyewitnesses of crimes, unlike observers of neutral events, recall details concerning the perpetrators’ actions immediately preceding the crime act better. Notably, individuals with lower scores on a scale measuring healthy emotionality (ESQ) demonstrate enhanced recollection for these details. At the same time, emotionality plays no significant role in recollection in repeated measurement, as well as in remembering the neutral event. The emotions experienced during crime observation appear to hinder the recollection of perpetrator appearance and behavior unrelated to the crime. These findings are discussed in light of the adaptive role of negative emotions in detecting danger and preparing for unpleasant stimuli.

## Introduction

Eyewitness testimony is often among the most important evidence used in court proceedings and treated by a court or jury on a par with biochemical traces left at the scene of a crime (Brewer and Wells, 2011). However, this crucial cornerstone in the edifice of criminal proceedings, heralded for its potential to shed light on the events of a crime, is not without its cracks. The intricate workings of human memory, susceptible to external and internal influences, cast doubt upon the reliability of eyewitness testimony. One such influence is emotion, whose impact on memory is still a subject of scientific debate.

Despite the considerable scientific inquiry into the influence of emotions on memory, the findings thus far have yielded a complex and sometimes conflicting landscape. Emotions, particularly negative emotions, have been found to enhance the vividness and strengthen the salience of memories (Todd et al., 2012, 2013; Markovic et al., 2014), what can be considered a factor enhancing their recollection observed in various research [e.g., Cahill and McGaugh, (1995), Vogel and Schwabe (2016), and Shields et al. (2017)]. At the same time, however they also negatively impact memory accuracy [e.g., Houston et al. (2013), Takahashi et al. (2006); for metanalysis see: Deffenbacher et al. (2004)]. The discrepancies in results may be a consequence of varied methodologies adopted, diverse ways of operationalizing emotions, or the complex and non-linear effects of arousal on cognitive processes [Lane and Houston (2021), Głomb (2022), and Snow and Eastwood (2022)]. However, another plausible explanation of the lack of consistency in experimental and field results is an insufficient consideration of individual variability in emotionality.

The investigation of individual differences in witness testimony is hardly a new concept. While initial studies primarily cantered around age, sex/gender, and race differences (Wells, 1978), knowledge regarding the influence of traits and predispositions in witness testimony has grown over time. Although the variables examined have predominantly encompassed factors that typically impair human cognitive functions, such as mental disorders (Soraci et al., 2017), regardless of the context of remembering.

Inter-individual differences observed in witness testimony can be also explained by emotionality. Emotionality encompasses the subjective experience of emotions, including their intensity, temporal aspects and feelings, as well as the individual’s capacity to control and regulate them, which can vary greatly across population. These variations can significantly influence the encoding, storage, and retrieval of information related to witnessed events.

Sparse attempts to examine the significance of emotionality to the performance of witness duties have yielded some promising leads suggesting that there is a link between the recollection of unpleasant and stressful events and various aspects of emotional functioning. For example, research points to the role of emotional intelligence, i.e., the ability to process, control, and understand the emotions, as a factor that promotes better memory of emotive events (Mikolajczak et al., 2009; Bagri and Galhardo, 2017). Some studies also suggest that anxiety can influence recollection of emotional events. For example, Dobson and Markham (1992) and Areh and Umek (2007) demonstrated that subjects low in neuroticism/anxiety are more reliable in recollection of details about a crime they witnessed. This is in line with results obtained by Siegel and Loftus (1978), which suggest that people who are anxious perform more poorly on a test measuring testimony accuracy. However, some research failed to replicate the results (Ridley, 2003; Curley et al., 2017), or even indicated that chronic trait anxiety promotes faster processing of threatening faces [e.g., Byrne and Eysenck (1995)]. This can be explained by an attention bias toward threating stimuli that results in enhanced memory to emotional details [e.g., Berggren and Eimer (2021)].

Therefore, it is evident that a consensus regarding the impact of emotionality on witness testimony is lacking, despite the theoretical plausibility of such associations. The considerable body of research that has been unable to establish the significance of individual differences in this regard – as predictors of performance and even as correlates of various aspects of testimony – implies that a meticulous and theoretically informed approach is imperative for their investigation.

In our view, the methodology which is difficult to compare between studies contributes to the problems of determining the role of emotionality in witness testimony. Research on individual differences relatively rarely seeks to identify and explore effects specific to eyewitness testimony. Moreover, a direct effect of individual variables on selected aspects of memory is frequently assumed, and the possibility that this relationship may not only be more complex but could also occur only under specific conditions is not considered enough. Inter-individual variation in traits and predispositions does not necessarily have a general effect on memory, but it may play a role in shaping the impact of emotions on cognitive processes when emotions are evoked. According to Trait Activation Theory [TAT, Tett et al. (2013)] traits are latent propensities which are expressed as responses to trait-relevant situational cues. This theory posits that specific situations can trigger or ‘activate’ particular traits, resulting in observable behaviors consistent with those traits. In the context of eyewitness testimony, emotional situations, such as witnessing a crime, could activate traits related to emotional disposition. Individuals with high emotionality might experience heightened emotional responses, thereby influencing memory encoding, storage, and retrieval processes. Thus, considering the dynamic interaction between individual emotionality and situational factors, such as stress during a crime, is crucial. This interplay may lead to varied eyewitness testimonies, as individuals with high emotionality may react differently to stress, danger, or other emotions compared to those with normal or reduced emotionality. Moreover, based on the theory, it can be assumed that we should not expect emotionality to be relevant in an emotionless context – or as Kenrick and Funder (1988) noted: “*Anxiety* (*…*) *shows up only in situations that the person finds threatening.*” (p. 4).

**Figure 1:**
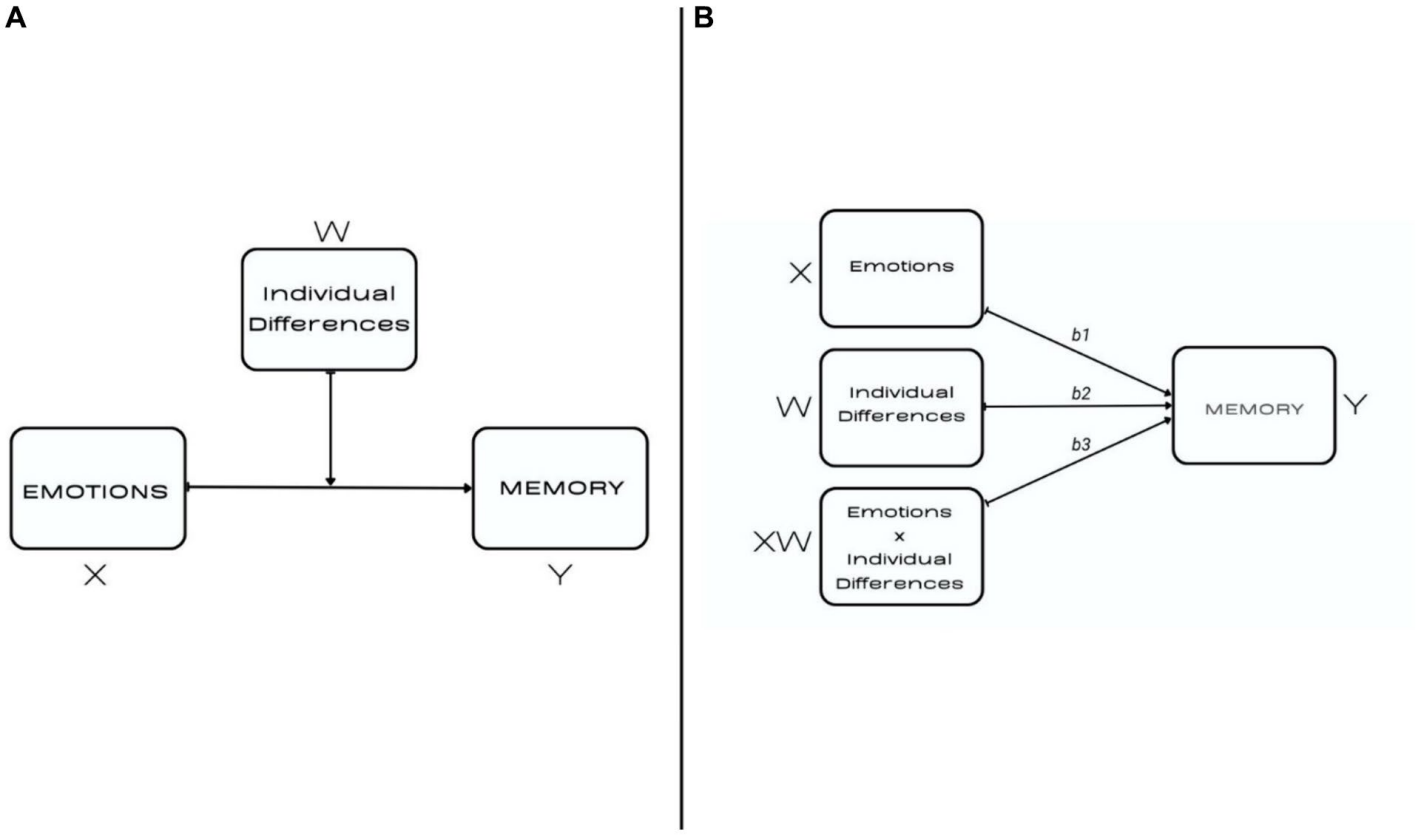
The basic interaction model tested in the study. **(A)** presents a conceptual diagram and **(B)** presents a formal statistical diagram. Based on diagrams proposed by Hayes (2018).

Hence, we argue that assessing the importance of witness characteristics in testimony requires employing a model that considers the interaction between conditions of remembering (such as emotional versus neutral) and individual differences in emotionality. Only this type of interaction can allow us to determine whether a given variable is relevant to a witness-specific context of memory formation, that is, rich in stimuli that evoke negative emotions. For experimental studies, moderation testing is an appropriate procedure, as it allows one to verify the assumption that the relationship between two variables (emotions → memory) depends on a third variable (emotionality). In other words, it is possible to establish the trait requirement for a causal relationship to occur. Moderation analysis extends beyond a simple comparison of main effects, i.e., the difference in remembering events of a different emotional nature, but also allows us to explain some of the variance in the results. Thus, we applied a basic moderation model presented in Figure 1 to analyze the data obtain in the experiment.

In this study, we also decided to go further than examining the role of traits reflecting the tendency to experience negative emotions, such as anxiety or neuroticism. Building on research on adaptive emotionality, we decided to explore individual differences relating not only to the ability to understand the emotions of others, but to regulate our own. Therefore, we turned to the relatively novel concept of healthy emotionality. It highlights the ability to achieve optimal biopsychosocial functioning rather than conceiving of health as a lack of pathology (Kesebir et al., 2020; Gasiorowska et al., 2022). A general predisposition to adaptive affective responses and effective emotional regulation is central to this concept and, thus, focal to this research.

Furthermore, we aimed to investigate the enduring impact of emotionality on eyewitness testimony over extended interval. Eyewitnesses typically provide initial reports to law enforcement promptly after witnessing an event, yet they may also be subjected to repeated inquiries throughout the investigation. Thus, we conducted repeated memory measurement to examine memory retention and the influence of emotionality as the emotional intensity of the witnessed event wanes.

In order to investigate the relationship between emotions (IV) and memory (DV), and the role of emotionality (Moderator) we designed a laboratory experiment with two conditions differing in the context of remembering: Criminal (presumed emotional) versus Neutral. The experiment aimed to compare: (1) the direct influence of emotion on the memory of individuals witnessing a crime; (2) the influence of emotionality on the emergence of this effect, and (3) the effect of time on the relationship between emotions, emotionality and testimony. Moreover, given that studies investigating the impact of emotion on memory functions sometimes suggest variations in the effects of stressful stimuli across different memory processes (e.g., Houston et al., 2013), we opted to analyze two specific aspects: the quantity of correctly recalled information using the free recall procedure (Event-recall memory) and its overall accuracy, as well as memory for faces investigated in eyewitness identification paradigm (Face identification).

Having this in mind, we have formulated specific hypotheses. First, [H1] we anticipate an effect of emotions on all memory measures. Aligned with the arousal-biased competition theory (ABC), which posits that arousal can influence information processing by directing resources to high-priority stimuli (Mather and Sutherland, 2011), we predict heightened cognitive mobilization in eyewitnesses experiencing negative emotions and arousal due to a criminal event, compared to those who observed a neutral event. Therefore, we hypothesize that [H1a] participants in the Criminal condition provide more correct details than those in the Neutral condition. Additionally, we expect [H1b] participants in the Criminal condition to outperform those in the Neutral condition in the identification procedure. Furthermore, we anticipate differences in overall accuracy between conditions (H1c), without presuming a specific direction for this difference.

Our main hypothesis, however, relates to the moderation of the relationship between the remembering context (criminal versus neutral) and memory for the event by healthy emotionality. It states that [H2] for witnesses of a crime, characteristics that reflect stable and healthy emotional functioning are beneficial. This assumption is in line with classic research pointing out the impact of neurotic traits on memory and task performance [e.g., Eysenck (1979)] as well as recent research on Emotional Intelligence (Bagri and Galhardo, 2017). In terms of our model it assumes [H2a] a significant interaction between condition if remembering and level of healthy emotionality. Specifically, we predict that [H2b] healthy emotionality promotes better remembering of a criminal event, while at the same time, [H2c] it plays no significant role in remembering neutral events. When negative emotions are not evoked, variables relating to emotional stability and adaptive affective responses are latent (as Trait Activation Theory assumes), and thus irrelevant. Therefore, we expect to seeno significant differences in memory performance between emotionally healthy and unstable individuals when they observe a neutral event.

We expect similar effects in the case of Accuracy (H3) as well as Face identification (H4), that is a significant interaction between condition and healthy emotionality, better performance in Criminal condition in case of participants with higher measures of this trait and no effect of emotionality in the Neutral condition.

Moreover, in terms of the repeated measurement of memory performance, we posit that as the intensity of experienced emotions diminishes over time, [H5] emotionality does not serve as a moderator of the relationship between emotions experienced during the event and memory measures.

## Materials and methods

### Participants

A total of 153 people participated in the experiment. Due to missing data, 150 participants (*f* = 96) were qualified for the final analyses of the first memory performance (Memory Task 1), and 124 for the second memory performance (Memory Task 2). In relation to Memory Task 2, 8 participants submitted their recordings with free recollection later than 7 days after the first measurement. Additionally, one person was excluded due to the low quality of the recording, which hindered transcription. The primary reason for the reduced sample size in the repeated measurement was participant attrition during the experiment, impacting 17 participants.

The study involved young adults (*M* = 22.1 years old; SD = 2.7). They were given monetary reward for their participation. The subjects were unaware of the real purpose of the experiment, and the memory tasks were not mentioned as part of the experimental instructions. When assigning to conditions in Neutral: *n* = 75; in Criminal: *n* = 75, similar female-to-male ratios were attempted in both groups.

Sensitivity analysis using G*Power (Faul et al., 2007) revealed that for linear multiple regression (fixed model, *R*^2^ increase) with power (1- β) = 80% and significance (*α*) = 0.05, and considering three predictors, a sample size of *n* = 150 is sufficient to detect an effect size of *f*^2^ = 0.052. While the effect size is small, it is considered adequate in this area of research. Previous studies have found similar effect sizes, with for example *β* values of approximately 0.20 or even less [e.g., Bagri and Galhardo (2017)].

## Materials and apparatus

### Experimental manipulation

For the study, we prepared two videos: criminal and neutral. The comparison of memory with comparable events is inspired by a similar procedure used by Houston et al. (2013). The basic premise was to prepare two films presenting scenarios that differ in the presence or absence of a criminal incident. However, they do not differ in terms of characters and the sequence of actions of the protagonists that lead to the crime. The scenarios used were staged events involving the same actors. The videos lasted about 3 min and presented a scene in a pub with an outdoor garden. The criminal incident involved two “Perpetrators,” male and female, who rob a female pub customer sitting next to them. To carry out the theft, the male perpetrator turns to the “Victim” and asks her for directions; at the same time, the female perpetrator approaches the table, takes a tablet and a wallet, and walks away from the scene. When the Victim realizes that her belongings have been stolen and tries to run after the female perpetrator, the male stops her by pushing her onto a chair and knocking the rest of the items off the table. In the control condition, the course of the scene was similar; however, it did not end with the theft, but with a conversation between the main characters (the same actors who played the Perpetrators) and the pub customer (the same actress who played the Victim), who helped them find their way^1^.

In both films, the characters are in the same place, at the same distance from the witness, sitting in the same poses, speaking about the same event, performing similar actions, and making similar comments about their surroundings. The videos, up to the point of theft, do not differ in any significant detail, which was later evaluated in free recall test. As our goal was to evoke negative emotions, in criminal condition we decided to present them as rude and unpleasant people. The difference in the presentation of the characters was primarily related to their body language (agitated moves and gestures), and the change in the tone of their comments, rather than additional behaviors and acts.

In the experiment, we used a 360-degree video, which participants watched using VR goggles. This type of video and the way it is played increases the field of view of the observer and allows them to see everything that happens around them. It also enhances the sense of being present in the scene. Consequently, it better simulates the experience of an actual eyewitness. The employment of this technology aligns with the methodological imperative to enhance the ecological validity of eyewitness testimony research (Yuille and Wells, 1991; Głomb, 2022; Kruse and Schweinberger, 2023). This method has been successfully used in other eyewitness research [e.g., Kloft et al. (2020)] and more broadly in the field of criminology [e.g., McClanahan et al. (2024)]. Moreover, the criminal video used in this experiment was tested in preliminary research, which suggested that this type of video enhances immersion, which is a feeling of being transported into or being part of the scene (Glomb et al., 2023).

### Memory performance

In this study, we analyzed two memory functions: recollection and recognition.

*Event-recall memory* is a measure that reflects episodic memory. It is the number of correctly remembered details about the event. Data taken into consideration included information on the course of the event and the perpetrators. It was developed after consultation with competent judges (researchers not involved in the project (psychologists) and a police officer). As events differed between conditions, we decided to include only details that were identical or similar in both groups. Therefore, this measure does not include details about the robbery itself, that is, what was taken from the victim, who took it, a description of the male’s behavior (throwing objects off the table and pushing the victim into a chair) and fleeing the scene of the crime. Additionally, we also included accuracy rate for free recall. This rate is defined as a number of accurately provided details of the event / Σ accurate + errors.

*Face identification* is a measure that reflects the accuracy of face identification performed by subjects. It consists of the sum of the recognized faces (range from 0 to 2), thus, it can be displayed as an average rate. Furthermore, we separately considered male and female faces, since the recognition accuracy may vary depending on the gender of the offender [e.g., Mason (1986)] and therefore on gender bias [see Herlitz and Lovén (2013) for meta-analysis]. For the purpose of the study, photo line-ups were created in accordance with the regulations and practices used by the police in Poland. The boards (see Supplementary material) showed pictures of the perpetrator’s face presented from the front along with faces of five decoys. The decoys, according to the regulations, were to be people of similar age and appearance, i.e., the same race and complexion, approximate hair color and hairstyle, and similar shape of facial features. Photos of the decoys were collected in a bank of faces prepared specifically for the project. Competent judges then selected from them 5 people who most resembled the perpetrators.

### Emotionality assessment

*ESQ-PL* (Gasiorowska et al., 2022) was used to assess healthy emotionality. It is a self-report measure that captures how people vary across six dimensions that make up a healthy emotional life: (1) *Outlook* captures an individual’s ability to sustain positive emotions and a general tendency to have a positive attitude; (2) *Resilience* refers to the temporal aspects of emotional response, but it captures the ability to disengage from negative affect; (3) *Social Intuition* is the degree to which a person is sensitive to social cues such as facial expressions, gestures, body language, or voice intonation; (4) *Self-awareness* is the ability to perceive body signals of emotions and to recognize and interpret them; (5) *Sensitivity to Context* reflects the ability to adjust reactions to the emotional and behavioral context of a situation; (6) *Attention* captures the ability to ignore distractions and not succumb to an attention-grabbing stimulus; (7) *Healthy Emotionality* (ESQ) is the overall score obtained in the questionnaire, it provides insight into the global predisposition to adaptive emotional responses and their regulation.

Subjects completed the ESQ, responding to 24 questions on a scale ranging from 1 (Strongly Disagree) to 7 (Strongly Agree).

### Additional measures

To ensure our experimental manipulation worked and evoked affective response, we asked subjects to rate the emotions the videos elicited. We also measured their electrodermal activity. Given that these measures serve as a manipulation check, thus, are of side interest, the results of the between-subjects comparisons are included in Supplementary material.

Moreover, during the filler tasks, participants completed a battery of questionnaires, including the HEXACO-60 (Ashton and Lee, 2009) and the Analysis-Holism Scale (AHS; Choi et al., 2007), both administered during Filler Task 1. In Filler Task 2, participants completed Kagan’s Matching Familiar Figures Test (Matczak and Kagan, 1992) on a computer. In this paper, our focus lies solely on healthy emotionality, measured by the ESQ, as it offers a novel contribution to research on emotionality. Therefore, the primary analysis does not center on the results of these additional measurements. However, the data collected during the experiments have been integrated into the repository’s database^2^. Additionally, within the Supplement (Additional Analysis D), we have included analyses concerning other measures of emotionality, specifically exploring the Emotionality domain and two facet-level scales assessed by the HEXACO-60. Given the interconnectedness between the concept of healthy emotionality and anxiety as a trait [e.g., Kesebir et al. (2020)], our study also investigates the relationship between these variables as part of an exploratory analysis.

### Procedure

The experiment was conducted in a between-subjects design. Subjects were randomly assigned to the condition at the time of enrolment. The conditions differed in the type of video presented: Criminal or Neutral.

The procedure included the following steps:

1. Baseline electrodermal activity measurement (neutral relaxing 360-degree video presenting the Milky Way).
2. Exposure to stimulus through VR goggles (Crime/Neutral 360-degree video) and measurement of electrodermal activity.
3. Emotions self-report.
4. Filler task 1. A battery of questionnaires which included ESQ-PL and additional measurements not discussed in this study.
5. Free recall Memory Task 1. Respondents were asked three questions: (1) *Tell all you remember about the scene in a pub that you just watched, both about how the scene unfolded and about the people who participated in it*. (2) *Do you remember anything about the appearance of the main characters*? (3) *Is that all you remember about the film?* The task format, i.e., including three questions, was developed after a pilot study which showed that subjects, when asked to describe “*everything they remember*” were limited to a very schematic and brief description of the events. As very short description do not allow for a reliable comparative analysis, we decided to expand the task and ask three questions. As our study is concerned with eyewitness testimony, the question about the perpetrators’ look was crucial. We also added a third question in case that a subject remembered something about the perpetrators’ behavior after recalling their appearance. Subject responses were recorded using a voice recorder and then transcribed and coded to be analyzed in terms of the amount of information provided.
6. Filler task 2 which involved Kagan’s MFF test administered on a computer. It was completed in approximately 4 min on average. However, the primary purpose of this task was not to serve as a time interval following Memory Task 1, but rather as a cognitively engaging distraction.
7. Face identification Test. The subjects were presented with an array of photographs of six faces [2×3 photographs] on a computer screen, one of which was the perpetrator’s face. Separate boards were prepared for the female and male perpetrator. Photographs were presented in a random arrangement. The instructions the subjects heard were as follows: “*Your next task is to recognize the faces of the people you saw in the video. Look at the pictures presented on the screen and point out the person that you remember seeing*.”

The time interval between the encoding memory and its retrieval in the first memory task (recollection) was set at 20 min.

8. Free Recall Memory Task 2. We asked participants to record their answers to the same questions as in task 1. The time interval between first and second task was set at 7 days.

The procedure was positively reviewed and approved by the Research Ethics Committee at the Institute of Applied Psychology at the Jagiellonian University before its application (decision number 56/2019 dated 25.11.2019).

## Results and discussion

### Step 1. Memory performance (task 1)

Prior the evaluation of the moderation model, we conducted a comparative analysis of the memory metrics investigated in the experiment, specifically focusing on Event-recall memory and Face identification.

In the case of Event-recall memory it was necessary to code and count the number of details correctly remembered by the group. Besides the overall recollection index (1), which includes details about the event, we also explored specific measures of recollection covering details blocked into domains corresponding to the perceptual fields. These are: (2) details about perpetrators’ look and behavior prior to the crime, together and by gender of the perpetrators, and (3) interaction with the victim (and her activity) that, in the criminal condition, resulted in robbery and assault, while in the neutral scene ended withpub customer helping two people to find their way. These two fields include, in our view, information that is temporally and geographically related; thus, it is believed that they are encoded at the same time. As mentioned before, we did not score the details of the theft itself, but only the information that was identical or highly similar across both conditions.

We then conducted between-subjects comparisons using t-tests on the data collected in Memory Task 1. As can be seen in Table 1, only overall Event-recall memory did not turn out to be significant [*t* (148) = − 1.361; *p* = 0.176]. However, when we take a closer look at the perceptual fields, we can clearly see between-subjects differences in recollection.

**Table 1:** Even-recall memory in Memory Task 1 Overall and in specific areas reflecting the thematic scope of information (*N* = 150).

Even-recall memory	Criminal *M* (SD)	Neutral *M* (SD)	Between-subjects comparisons *t* (148)
Overall all details	14.19 (5.48)	15.39 (5.32)	−1.361; *p* = 0.176;
Perpetrators look and behavior	10.24 (4.58)	12.60 (4.89)	−3.051; *p* = 0.003; *d* = 0.498
Male perpetrator	5.52 (2.64)	6.68 (2.81)	−2.60; *p* = 0.010; *d* = 0.425
Female perpetrator	2.44 (1.65)	3.31 (1.99)	−2.90; *p* = 0.004; *d* = 0.473
Interaction with ‘Victim’	3.45 (1.67)	2.11 (0.95)	6.064*; *p* < 0.001; *d* = 0.990

*Due to the violation of equal variation assumption a Welsh *t*-test with Satterthwaite approximation for the degrees of freedom was used.

Subjects who watched neutral event remembered details about the look and behavior of the perpetrators before the criminal act significantly better – both overall [*t* (148) = −3.051; *p* = 0.003; *d* = 0.498], and by gender of the perpetrator [for male: *t* (148) = −2.60; *p* = 0.010; *d* = 0.425; for female: *t* (148) = −2.90; *p* = 0.004; *d* = 0.473]. However, after applying the Benjamini–Hochberg procedure (Benjamini and Hochberg, 1995) for controlling multiple comparisons, we found that the comparison of the male perpetrator is no longer significant once the correction is applied (see Supplementary Table B1). Eyewitnesses of criminal event, on the other hand, remembered more correct details about the interaction between the perpetrators and the victim [*t* (148) = 6.064; *p* < 0.001, *d* = 0.990]. They gave more accurate information about the part of the scene that immediately preceded the crime.

In addition to comparing the number of correctly remembered details, we also performed an analysis of the accuracy of the testimony. The accuracy rate was calculated as a number of accurately provided details of the event / Σ accurate + errors – similar to research of Evans and Fisher (2011). As can be seen in Table 2, the rate was found to be significantly lower for the group witnessing the crime [*t* (148) = −3.865, *p* < 0.001; *d* = 0.631]. Thus, while in general, the number of correctly remembered details does not discriminate between conditions, it is the ratio of correct and incorrect information that differentiates them in favor of neutral condition.

**Table 2:** Accuracy rates of testimony across conditions (*N* = 150) for Memory Task 1.

	Condition	M	SD	SEM
Accuracy (all details)	Crime	0.887	0.0861	0.0099
	Neutral	0.934	0.0605	0.0070
Between-subjects comparison	*t* (148) = −3.865, *p* < 0.001; *d* = 0.631			

We also examined Face identification rates. As can be seen in Table 3, subjects in both conditions identified the female perpetrator almost equally well [*χ*^2^ (1, 150) = 0.244; *p* = 0.621]. They also did not differ in general accuracy of recognition of both faces. However, we obtained different results for the male perpetrator. Eyewitnesses of the crime performed significantly worse than subjects observing neutral event [*χ*^2^ (1, 150) = 6.07; *p* = 0.014; *eta* = 0.201]. Thus, we can conclude that the face of the man was remembered worse, resulting in more misidentifications in a procedure resembling a police line-up.

**Table 3:** The line-up identification results (*N* = 150) and general face identification rate based on average recognition of two perpetrators.

RM	Criminal			Neutral			Between-subjects comparisons
	Hit	Miss		Hit	Miss		*χ*^2^ (1, 150)
Male perpetrator	26	49		41	34		**6.07; *p* = 0.014; *eta* = 0.201**
Female perpetrator	44	31		41	34		0.244; *p* = 0.621
General	Number of hits						4.325; *p* = 0.115
	0	1	2	0	1	2	
	22	36	17	21	26	28	
	Recognition rate						*t* (148)
	0.47			0.55			−1.278; *p* = 0.203

Bolded values indicate significant results.

Concluding this step of the analysis, we revealed that eyewitness recollection focused on those details that were directly related to the behavior of the protagonists immediately before the crime. At the same time, eyewitnesses of the crime remembered the perpetrators worse. Not only did they give significantly fewer details about the perpetrators’ look and behavior prior interaction with a victim, they also tend to make more mistakes during identification of male perpetrator. Moreover, the accuracy rate of testimony was significantly lower for individuals witnessing the crime. The results, thus, suggest differences in the recollection of a criminal and neutral event, but the direction of these differences depends on the specific content of memory.

### Step 2. Testing the moderation model

The main goal of our research is to investigate the role of healthy emotionality in shaping the impact of emotions on memory. We performed a series of moderation analyses using PROCESS macro v.4.1 (Hayes, 2012). The outcome variable for analysis was one of the measures of Event-recall memory (overall and broader specific); the focal predictor of analysis was the research condition (criminal versus neutral), and, therefore, the absence or presence of negative emotions. The moderator variable evaluated for analysis was overall score of ESQ.

Quantitative variables were standardized prior to analysis, while the qualitative variable (condition) was cantered. Johnson-Neyman output was selected to assess when the effect of X on Y ceases to be significant.

As can be seen in Table 4, moderation analysis showed no significant effect of the healthy emotionality (ESQ) on overall Event-recall memory measure and for details concerning the perpetrators. However, we obtained a significant interaction between condition and emotionality when it comes to the third Event-recall memory measure: details related to the interaction between the perpetrators and the victim. The relationship between emotions and this memory measure was significantly moderated by overall ESQ score (*coeff* = 0.311; *p* = 0.034). The model explained 23.4% of the variance in recollection (Table 5). Thus, the results provide evidence supporting the validity of Hypothesis 2a.

**Table 4:** A summary of the moderation analysis (*N* = 150) performed on standardized variables in Memory Task 1.

		**Event-recall memory**		
ESQ* X		General	‘Perpetrators’ Look and behavior	Interaction with ‘Victim’
	*β*	0.065	−0.050	**−0.155**
	se	0.079	0.078	**0.072**
	*t*	0.813,	0.645	**−2.142**
	*p*	0.417	0.520	**0.034**
	*95% Cl*	−0.222; 0.092	−0.204; 0.104	**−0.299; −0.012**
			**Accuracy**	
		*β*	0.021 0.018 1.146 0.253 −0.0149;0.056	
		se		
		*t*		
		*p*		
		95% Cl		
			**Recognition Rate**	
		*β*	0.137	
		se	0.189	
		*t*	0.724	
		*p*	0.471	
		95% Cl	−0.237; 0.510	

Bolded values indicate significant results.

**Table 5:** Results of the moderation analysis (*N* = 150) performed on centered antecedent (Condition) and standardized moderator (ESQ) and outcome variables.

	Coeff.	SE	*t*	*p*
Constant	0.000	0.072	0.000	1.000
Condition (X)	0.445	0.072	6.159	0.000
ESQ (W)	−0.098	0.072	−1.357	0.177
Interaction Condition x ESQ (XW)	−0.155	0.072	−2.142	0.034
*R^2^* = 0.234; *MSE* = 0.782				

The outcome variable is Event-recall memory related to details about the interaction between the perpetrators and the victim in Memory Task 1.

**Figure 2:**
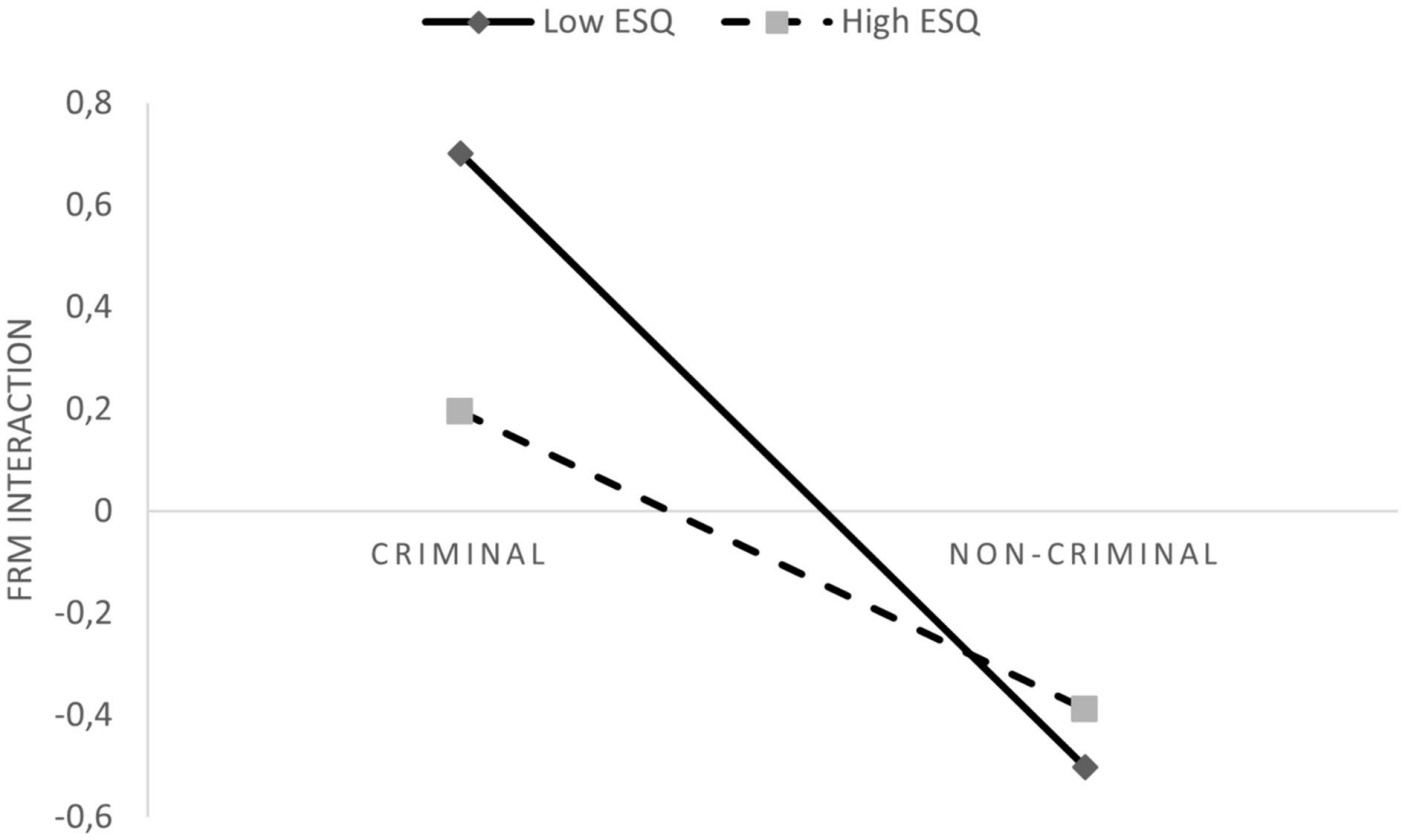
Interaction plot depicting the effect of healthy emotionality (ESQ) on relationship between emotions and Event-recall memory related to interaction between perpetrators and victim.

Examination of the interaction plot (Figure 2) showed that, when negative emotions are present, we can expect better memory performance at a low level of ESQ. At the same time, at a high level of healthy emotionality the number of correctly recalled details is lower. These results contradict Hypothesis 2b. Moreover, the difference in recollection of details about the interaction between low and high scorers in overall ESQ score is insignificant when the recalled event is non-criminal, and does not trigger stronger negative emotions. This aligns with Hypothesis 2c.

We also performed moderation analysis for Face Identification; however, the recognition rate did not turn out to be moderated by ESQ. The same is true for the accuracy rate for which we observed only the direct effect of emotion, not the indirect effect of emotionality. The summery of those two models are presented in Table 4. Thus, Hypotheses 3–4 were not confirmed.

Thus, as our hypothesis are concerned, we obtained results that, on the one hand, confirm the interaction effect of healthy emotionality on one of the memory measure, but, on the other hand, indicate the opposite sign of moderation. An emotion inducing criminal event is better remembered by people who are characterized by low scores on healthy emotionality, which corresponds to a tendency to experience negative affect for a long time, lower competence in understanding one’s own and others emotions, and succumbing to strong distracting stimuli.

### Step 3. Repeated measurement of memory performance

Table 6 illustrates the between-condition comparisons of the data derived from Memory Task 2. Notably, a significant difference is observed in the number of correctly recalled details concerning the interaction between the Victim and Perpetrators [*t* (107.02) = 1.80, *p* < 0.001; *d* = 0.923]. Furthermore, a trend is observed, suggesting better recollection of details about the female Perpetrator in the neutral condition [*t* (122) = 1.81, *p* = 0.072; *d* = 0.326].

**Table 6:** Event-recall memory in Memory Task 2: overall and in specific areas reflecting the thematic scope of information (*N* = 150).

Event-recall memory	Criminal *M* (SD)	Neutral *M* (SD)	Between-subjects comparisons t (122)
Overall (all details)	12.02 (5.22)	11.17 (5.22)	0.898; *p* = 0.371
Perpetrators look and behavior	8.20 (4.21)	8.83 (4.84)	−0.771, *p* = 0.442
Male perpetrator	2.34 (2.05)	2.32 (2.48)	0.065, *p* = 0.948
Female perpetrator	1.41 (1.81)	0.92 (1.13)	1.813, *p* = 0.072; *d* = 0.326
Interaction with ‘Victim’	3.46 (1.72)	2.10 (1.20)	1.800*, *p* < 0.001; *d* = 0.923

*Due to the violation of equal variation assumption a Welsh *t*-test with Satterthwaite approximation for the degrees of freedom was used.

Given that in first measurement of memory performance emotionality only significantly affected the recollection of details concerning the interaction between the victim and the perpetrators, we opted to test the moderation model, including emotions (IV), emotionality (Moderator), and these specific details (DV). Results revealed a significant overall model (*R* = 0.43, *R*^2^ = 0.19, *F* = 9.11, *p* < 0.001), indicating that the combined effects of condition and emotionality significantly predicted memory performance. Specifically, participants in the criminal condition exhibited significantly higher scores compared to the neutral condition (*coeff* = −0.834, *p* < 0.001). However, emotional state did not significantly moderate this relationship (*coeff* = −0.233, *p* = 0.347). The interaction was also not significant (*coeff* = 0.1167, *p* = 0.465), suggesting, in accordance with our hypothesis, no significant effect of emotionality on repeated memory measures.

**Figure 3:**
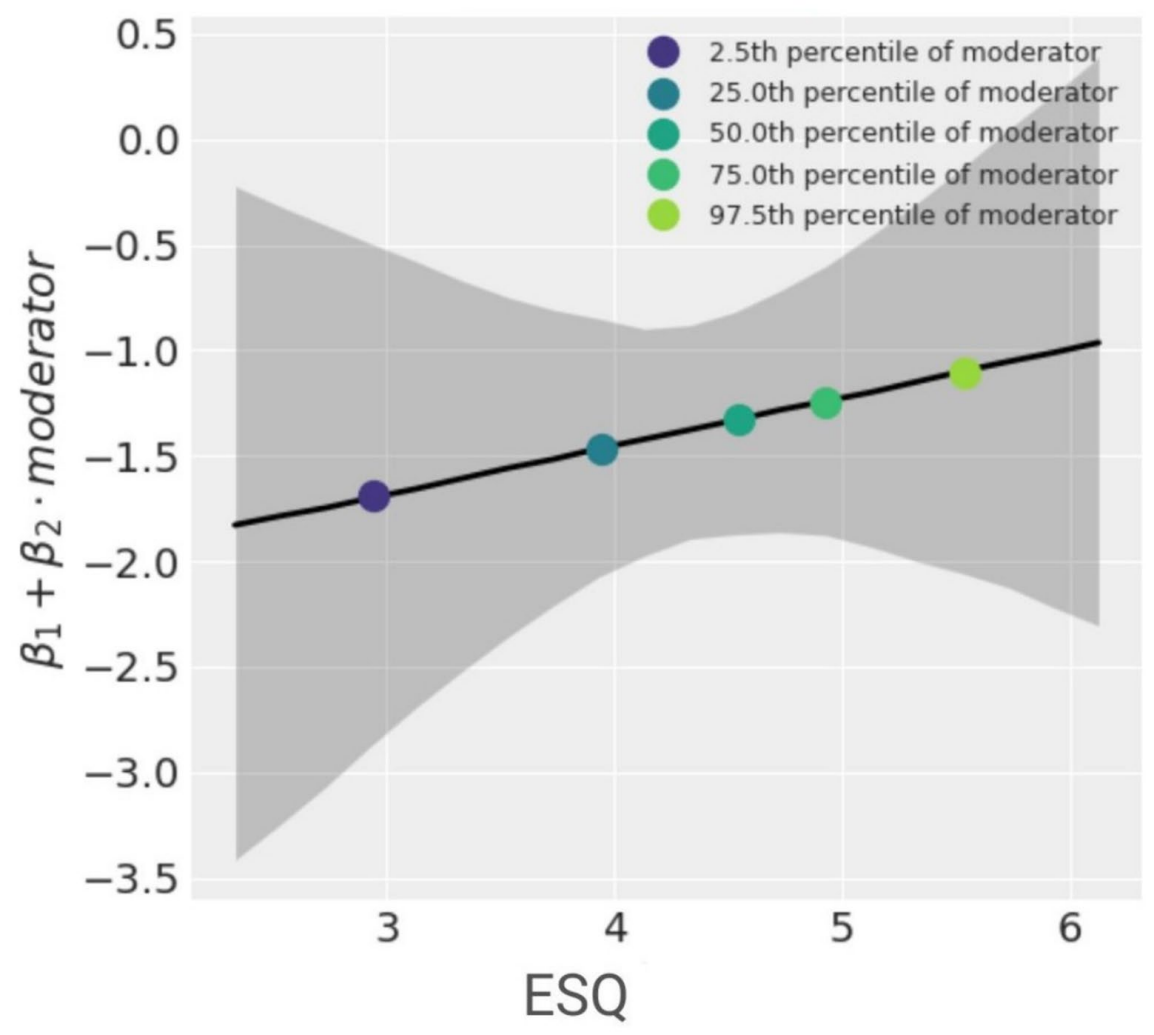
Spotlight graph made for Bayesian moderation analysis (Free Recall Memory Test 2) depicting the rate of change of the outcome (number of correctly remember details about interactions between victim and perpetrators) per unit of *x*.

However, as the hypothesis regarding moderation in Memory Task 2 presumed no interaction between condition and emotionality, we repeated the analysis using Bayesian moderation analysis. We employed a Python procedure proposed by Vincent (2023). The results confirm the findings provided by classical hypothesis testing.^3^ The mean value of the interaction parameter (*β*^2^) was estimated to be 0.23. However, the 94% highest density interval (HDI) ranged from −0.47 to 0.91, indicating considerable uncertainty in the estimate. This suggests that there is no clear evidence of a significant interaction effect between the moderator and predictor variables. This is further visualized in the spotlight graph (Figure 3) which shows the rate of change of the outcome (memory) per condition. It demonstrates that as the ESQ score (the moderator) increases, the number of recalled details about the interaction does not change significantly. These results confirm Hypothesis 5, indicating no moderation effect of emotionality after a time delay.

## General discussion

The purpose of our study was to investigate the relationship between emotions, emotionality and memory, with particular focus on the memory of criminal events. The results of our study suggest impaired memory performance for details related to perpetrators look and behavior (unrelated to crime itself), but at the same time improved memory for details related to interaction between perpetrators and victim (prior the criminal act). In addition the testimony of witnesses to a crime is generally less accurate, they are also more likely to misidentify the male perpetrator from those who observed a neutral event. The study also revealed the importance of individual differences in emotionality for some measures of memory. Emotionally unstable (the opposite of healthy emotionality) individuals remember the moment of the event when the perpetrators made contact with the victim better. However, this finding holds true only when witnesses provide testimony shortly after the emotional event occurs, suggesting a time-sensitive relationship. Notably, emotionality appears to have no bearing on the recollection of a neutral event.

In general, we found no difference in the amount of correctly recalled information between witnesses to a crime and observers of a neutral event. However, recollection strategies vary depending on whether one is confronted with a crime or a neutral event.^4^ In the case of a criminal event, details that immediately preceded the culminating event, i.e., the act itself, were more salient. Drawing on arousal-biased competition theory which states that arousal can affect information processing by directing resources to high-priority stimuli (Mather and Sutherland, 2011), it can explain the lower number of details concerning those parts of the event that were less relevant to the crime. However, to be in line with the assumptions of the theory, we should also observe a better memory of the perpetrator’s appearance, both in its description and recognition. Meanwhile, the eyewitnesses to a crime did significantly worse than individuals who watched neutral event when identifying the face of the male perpetrator. Furthermore, in-depth analysis of the details remembered (taking into account only information on the look of the perpetrators, see Supplementary material), suggest that there is a trend toward poorer recollection of the male perpetrator in the case of witnesses to a crime. Thus, it seems that the appearance of the violent perpetrators may be particularly sensitive to the effects of negative emotions.

The most important goal of our study, however, was to examine the role of healthy emotionality regarding the memory of witnesses. Although we did not reveal the interaction between condition of remembering and overall witness performance, there was a significant moderation effect for details related to the interaction between perpetrators and victim. The role of emotionality was revealed in relation to the crucial moment of the event that led to the crime; thus it is in line with trait activation theory. People who scored low in healthy emotionality, while recalling the event, paid special attention to details crucial, from the point of view of eyewitness role in criminal proceeding.

The study may therefore suggest that individuals with these characteristics may be particularly sensitive to emotional or even threatening stimuli. This trait may be potentially adaptive in nature – allowing one to respond more quickly and effectively in the face of danger (Marks and Nesse, 1994). Possibly, scanning the perceptual field for negative stimuli may somewhat resemble the strategy used by individuals who exhibit traces of defensive pessimism. They may use this tactic to be better prepared for negative emotion, thus gaining a feeling of control, and using the anxiety as a motivation for better performance (Cantor and Norem, 1989). Such a strategy can be effective as long as one does not face stimuli that are so strong that they disorganize cognitive processes. Therefore, we expect that negative emotionality fosters better recollection in witnesses who are at a relatively safe distance from the crime and are not directly threatened by it.

Our results can also be related to individual differences in anxiety. Initial validation study of the ESQ (Kesebir et al., 2020) have demonstrated a negative correlation between healthy emotionality and variables associated with anxiousness and stress. Additionally, our exploratory analyses revealed some overlap between healthy emotionality and Anxiety and Fearfulness, as well as the broader domain of emotionality measured by the HEXACO-60 (see Supplementary material). Therefore, the results of our study also appear to be in line with the findings of Burke and Mathews (1992), who demonstrated that anxious subjects recall more anxiety-induced memories and recall them faster, compared to individuals who do not suffer from anxiety disorder. This may suggest that people characterized by negative emotionality prioritize negative stimuli – threatening events are more readily recalled for an anxious individual.

Taking into consideration the results of repeated measurement of memory performance and no significant impact of emotionality, it is plausible that this prioritization, however, is a relatively unsustainable and time-sensitive effect. That is, in a situation where emotions are subdued, and testimony is provided after a period of time following the observation of the event, witnesses with both healthy and unstable emotionality recall a similar number of details accurately. This finding lends support to Trait Activation Theory, which posits that traits only manifest significance within their characteristic context.

Although our findings do not contradict the body of knowledge about individuals characterized by negative emotionality in general, they do yield results different from the aforementioned research on the importance of anxiety in witness testimony. It seems that this contradiction may be due to the different methodology used in our study. What was crucial for us was to demonstrate the importance of emotionality specifically for eyewitnesses; thus, we were searching for the interaction between the type of event remembered and memory not a prediction of memory performance based on individual differences. It is possible that the few studies that have shown the role of anxiety on memory [e.g., Siegel and Loftus (1978) and Dobson and Markham (1992)] have revealed its relevance to task performance, regardless of the context of remembering.

### Limitations and future research

We believe that our study has the potential to reinvigorate the discussion of emotionality and related areas of individual differences in the accuracy of witness testimony. However, it is not free from limitations. First, the study is based on only one experiment, which allows inferences limited only to the crime presented. We are uncertain whether similar trends will be observed for crimes that involve greater aggression and harm to the victim. It is possible that if the victim had not been a young woman (thus resembling the group studied, and therefore potentially more easy to identify with), we would have obtained different results.

The strength of our inference is also limited by the fact that, although we made every effort to present videos with similar sequences to the subjects under both conditions, there are obvious differences between them. Thus, we must consider that it was factors other than the presence of the crime that triggered the negative emotions and that it was not the emotions that determined the difference in recollection. However, as evident by the manipulation check regarding subjective rates of emotions and objectively measured arousal evoked by the videos (see Supplementary material), we have a firm reason to believe that the subjects actually felt negative emotions more strongly in the Criminal condition. Furthermore, in qualitative analyzing the reports of the participants, we saw a noticeable difference in the emotional overtones of their testimonies. Subjects in the Criminal condition often described their own mindsets toward perpetrators, judging their behavior and attitude negatively. Thus, we have a sense that it was emotions – though not necessarily just simple emotions, as measured in our study, but also more complex ones (e.g., moral emotions) – that differentiated the conditions. Moreover, as these scenes did not differ in regard to the number of details presented, we believe that differences in memory performance are not the consequences of different range and variety of stimuli.

Regarding future research directions, in addition to the essential replications, in other age groups (in the case of subjects, perpetrators, and victims) and varied types of crime, it seems crucial to test the potential applicability of the results in practice. The knowledge of the relationship between emotionality and witness testimony can be used by law enforcement to better prepare for testimony collection and facilitate better recall. Thus, testing appropriate interventions that can increase the amount and accuracy of recalled information for witnesses with a particular emotional profile appears to be an important research direction.

## Data availability statement

This research is part of a larger project aimed at investigating the impact of individual differences on eyewitness testimony. Data supporting this project are available at: https://osf.io/qrdk7/. The repository also includes a file with description of all measurements taken in this experiment. Please note that current study present only part of the data collected.

## Ethics statement

The studies involving humans were approved by the Research Ethics Committee at the Institute of Applied Psychology at the Jagiellonian University. The studies were conducted in accordance with the local legislation and institutional requirements. The participants provided their written informed consent to participate in this study. Written informed consent was obtained from the individual(s) for the publication of any potentially identifiable images or data included in this article.

## Author contributions

KG: Conceptualization, Data curation, Formal Analysis, Funding acquisition, Investigation, Methodology, Project administration, Visualization, Writing – original draft, Writing – review & editing. PP: Methodology, Supervision, Writing – original draft, Writing – review & editing. BG: Methodology, Supervision, Writing – original draft, Writing – review & editing. IR: Formal Analysis, Visualization, Writing – original draft, Writing – review & editing. MM: Investigation, Writing – original draft, Writing – review & editing.

## Footnotes

^1^For convenience, terms such as “Perpetrators” and “Victim” will be used for both the Crime and Neutral condition.

^2^For further information see Data Availability Statement.

^3^A Python-based procedure for conducting Bayesian moderation analysis, suitable for application on our dataset, has been included in the files available at OSF repository.

^4^Additional confirmation of the significant differences in recall of details about each perceptual field is provided by the additional analysis presented in Additional Analysis B (Supplementary Appendix A), which was designed to counter the problem of multiple comparisons. It sustained the significance of differences in all Event-recall memory measures relevant to the main analysis, except for the difference in recalling information about the look and behavior of the male perpetrator.
