# Supplementary material for: How emotions shape memory? The moderating effect of healthy emotionality on eyewitness testimony
Source: Front Psychol. 2024 Jun 6;15:1406897. doi: 10.3389/fpsyg.2024.1406897 (PMC11189012; doi:10.3389/fpsyg.2024.1406897)
Supplement: Supplementary file 1 [file Data_Sheet_1.pdf]

Supplementary Material

Methods and Material

Video. The videos were shot using a GoPro Fusion camera, which allows to produce high quality 360-degree videos. To present them we used HP Reverbs G1 goggles (head-mounted device) and HP Omen laptop.

Photo line-up. The boards showed pictures of the perpetrator's face presented from the front along with faces of five decoys. Photos of the decoys were collected prior the research in a bank of faces prepared specifically for the project. Competent judges then selected from them 5 people who most resembled the perpetrators.

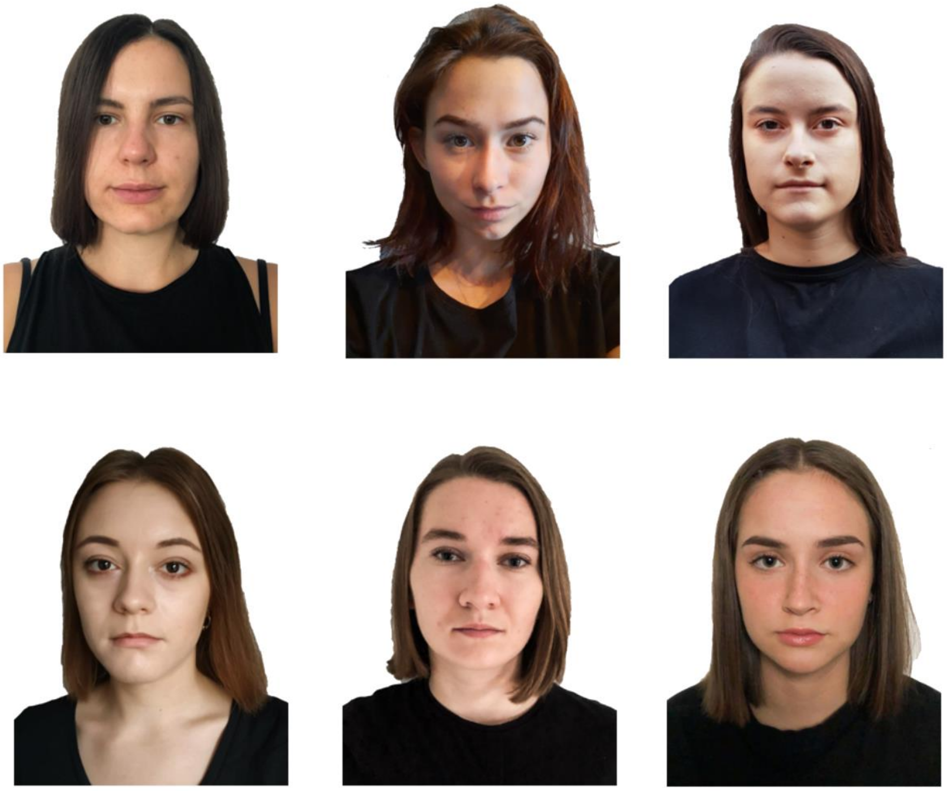

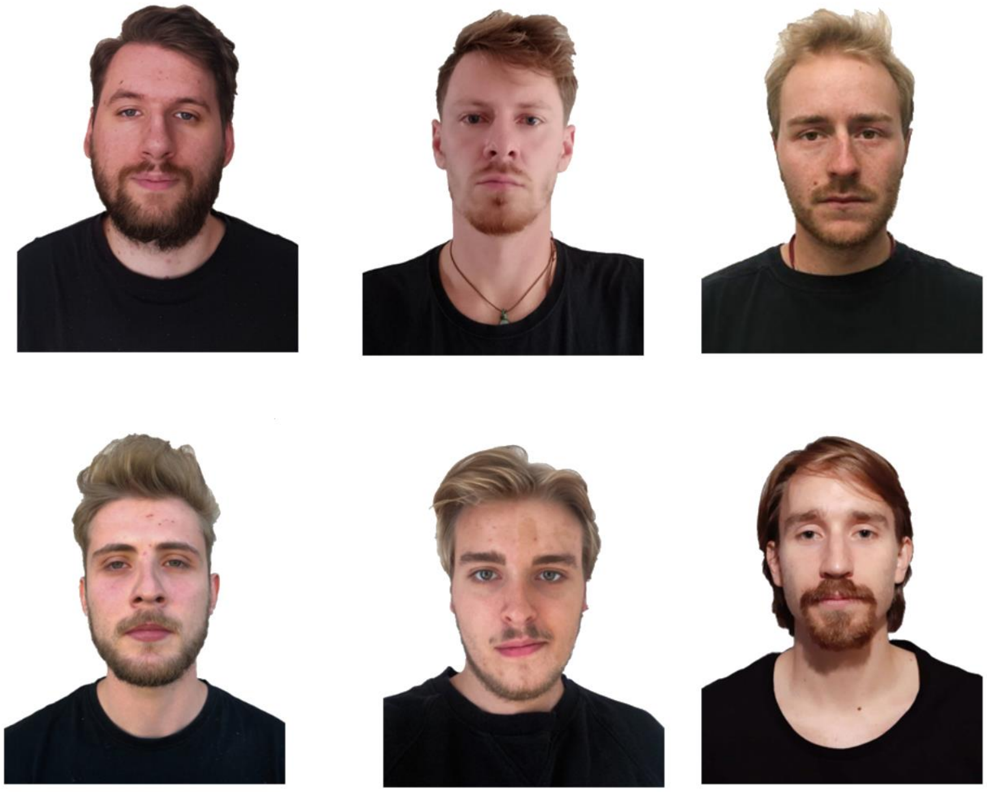

Additional Analysis A: Manipulation Check

To confirm the effectiveness of our experiment in terms of evoking emotions, we administered two manipulation checks reflecting two component of emotional reaction: arousal and subjective feeling.

Arousal. In this study, electrodermal activity (EDA) was measured. We used a wireless Shimmer3 GSR+ unit (worn as a wristband on the non-dominant hand) and two EDA diodes attached to the middle and index fingers. The unit was calibrated with a maximum sampling rate frequency of 1092 Hz. To reduce noise interfering with the measurements, the subjects were asked to take a comfortable position, place their forearms on the desk, and attempt to minimize hand movement while watching the video.

EDA analysis began with an initial review of the data for any measurement irregularities that may have been caused by equipment dysfunction or incorrect diode insertion. As a result, two records (one per condition) were eliminated.

Considering the procedure we applied, namely exposure to the film, which was designed to elicit general arousal not necessarily related to a specific, single stimulus, we analyzed the Skin Conductance Response (SCR) based on the frequency of peaks recorded in fixed time-periods (Braithwaite et al., 2013). In order to eliminate inter-individual differences in electrodermal activity, we also decided to compare the measurements recorded when subjects were watching the Crime/Neutral video with the measurement collected at the beginning of the study, when subjects watched a neutral, relaxing film (baseline). We chose two segments lasting 165 seconds for the comparison. We did not consider the first several seconds of the videos in order to eliminate the initial arousal associated with the novelty effect of the stimulus.

The extraction of tonic and phasic EDA components was performed based on the methodology proposed by Hossein Aqajari et al. (2021). The data were then checked for possible

abnormal readings and outliers suggesting interference during data collection, significant movements and other possible artifacts. As a result, two records per condition were eliminated. Thus, 144 records (72 per condition) were included in the final analysis.

Table A1 presents the results of the electrodermal analysis. To compare the arousal between-subjects, we conducted independent t-tests for two samples. First, we compared the absolute number of amplitudes recorded in subjects while watching the film ($t(142) = 1.713$; $p = .088$, $d = .286$). Second, we compared the difference in the number of peaks between the film and the baseline ($t(142) = 2.669$; $p = .008$, $d = .445$). The results indicate that the change in arousal between the first and second measurements was significantly higher for the Criminal condition. Further supporting this claim is the filtered measurement of electrodermal activity presented in Figure A1, which indicates higher skin conductance values in this condition. A similar course of arousal, with higher amplitudes at certain points in the film, reflects discrete differences in the presentation of the characters, who in the Criminal condition were expected to make an impression as more unpleasant that in neutral condition.

Table A1

Results of electrodermal activity analysis (N = 144)

		Criminal<br>n = 72		Neutral<br>n = 72	
		M (*SD*)	*Min - Max*	*M* (*SD*)	*Min - Max*
Number of peaks	Baseline	13.36 (1.60)	9 – 17	13.75 (1.12)	11-17
	Film	13.51 (1.54)	10 – 16	13.10 (1.38)	10-16
	Difference	.15 (2.04)	-4 – 6	-.65 (1.55)	-5 – 2

Figure A1

Filtered electrodermal activity recorded while watching the video. Between-subjects comparison

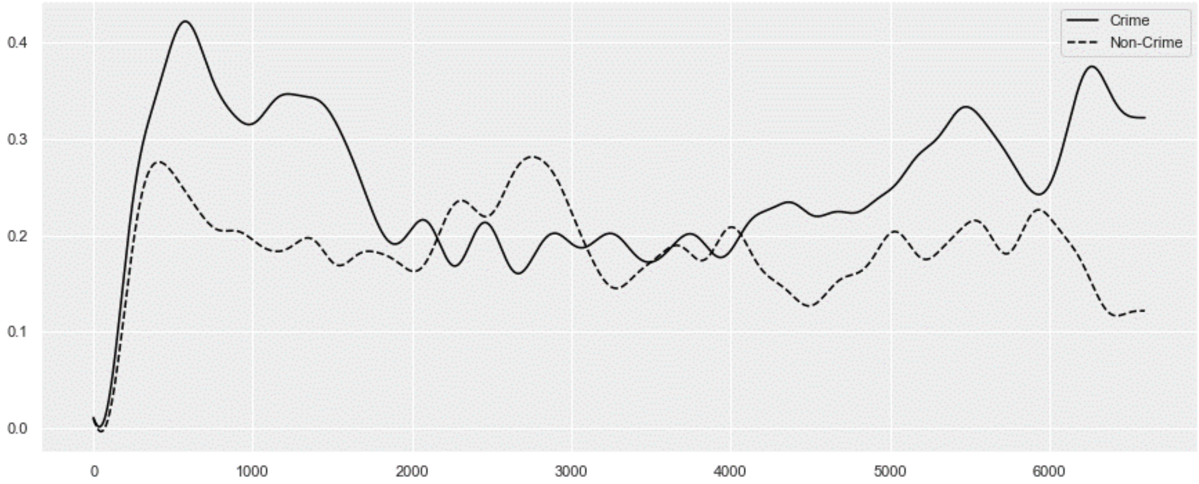

Subjective feeling. We used the Geneva Emotion Wheel (Sacharin et al., 2012) to determine the valence and intensity of emotions experienced by respondents while watching the film. It is a self-report measure consisting of discrete emotion labels corresponding to emotion families that are arranged in a circle divisible into four quadrants. The alignment of emotion terms is fundamental to the two-dimensional space limited by the axis of valence (negative to positive) and control (low to high). As a result, the method allows us to measure four domains of emotions (negative/low control, negative/high control, positive/low control, and positive/high control). The response options correspond to different levels of intensity for each emotion family, from low intensity (1) to high intensity (5). Subjects can also indicate that they did not feel a particular emotion (0) and they can independently label the name of the emotion they experienced. The final scores of these four domains are calculated as the mean rating values obtained for all discrete emotions that comprise these domains.

Immediately after watching the video, participants were asked to identify discrete emotions and rate the intensity they felt while watching the video. To answer the question about the difference in emotional response between witnessing a crime scene and a neutral scene, we created a negative emotion (NE) index, which is the average score of all 10 discrete emotions available on the GEW with negative valence (Anger, Hate, Contempt, Disgust, Fear, Disappointment, Shame, Regret, Guilt, and Sadness). Additionally, we counted two additional indexes that differentiate negative emotions on the dominance dimension, which allows more detailed conclusions about the relationship between the type of crime and witness reactions. As a result, we were able to compare the low-control (Disappointment, Shame, Regret, Guilt, and Sadness) and high-control (Anger, Hate, Contempt, Disgust, and Fear) negative emotions between subjects. We also counted the positive emotions (PE) index as an additional manipulation check This is the average intensity of all the positive emotions experienced while watching the film.

To compare the emotional reactions of witnesses to a crime and those observing a neutral scene, we performed Student's t-tests for independent samples. The results are presented in Table A1. Participants who observed the crime event felt stronger negative emotions – in general and both types of negative emotions in terms of dominance (high and low) – than those who watched the similar neutral event. Moreover, the results suggest no difference in positive emotions ratings between subjects.

Table A2

The average results of the emotion intensity ratings in each study condition and the between-subjects comparison (N = 150)

Emotion	Criminal	Neutral	Between subjects comparisons
	M (SD)	M (SD)	t(148)

NE	1.22 (.91)	.22 (.37)	8.84*; $p < .001$, $d = 1.44$
NE high	1.46 (1.07)	.24 (.51)	8.90*; $p < .001$, $d = 1.45$
NE low	.99 (1.05)	.20 (.35)	6.15*; $p < .001$, $d = 1.03$
PE	.93 (.69)	.97 (.80)	.35; $p = .727$

Notes: * due to the violation of equal variation assumption a Welsh t-test with Satterthwaite approximation for the degrees of freedom was used.

NE – Negative Emotion index; NE high – Negative Emotion with high control index; NE low - Negative Emotion with low control. PE – Positive Emotion index.

Additional Analysis B: Multiple Comparisons in Event-recall memory

In our study, we compared multiple measures of memory performance. *Multiple comparison problem* also known as *multiple testing problem* is a well-known statistical issue occurring when a wide range of inferences are tested at the same time which can lead to an increased risk of false positive results. To counteract this issue, we use a correction for multiple comparisons, the Benjamini–Hochberg procedure. It is considered to have more power than the alternatives - the Bonferroni and Sidak procedures - when more than three comparisons are made (Benjamini and Hochberg, 1995). This stepwise procedure sorts the obtained p values from lowest to highest and compares them to a critical value $(i/m)Q$, where i is the rank, m is the total number of comparisons, and Q is the False Discovery Rate. According to the procedure, the largest p value that is smaller than $(i/m)Q$ is significant, and all of the p values smaller than $(i/m)Q$ are also significant. Table S3 shows the results of the Benjamini–Hochberg correction with the false discovery rate set to 10%. Once the correction is applied (see Table B1), the comparison of the male perpetrator is no longer significant. All other measures (Interaction, Perpetrators look and behavior and Female perpetrator) remain significant.

Table B1

Results of the analysis with the Benjamini-Hochberg correction for multiple comparisons.

Memory performance	*p*	Rank	Critical Value (i/m*Q)
**Interaction with ‘Victim’**	**<.001**	**1**	.02
**Perpetrators look and behaviour**	**.003**	**2**	.04
**Female perpetrator**	**.004**	**3**	.06
Male perpetrator	.010	4	.08

Overall (all details)	.176	5	.10

Additional Analysis C: Memory of Perpetrators Look

In the main study, we analysed the behaviour and look of the perpetrators as a single domain. In our view, this type of information constituted a common perceptual field – at the beginning of the scene, perpetrators' look and actions constitute a single figure (Gestalt) against the background, which is the rest of the pub and its attendees. However, it is possible to frame the scene in such a way that the appearance of the two perpetrators is taken into account separately. Below are the results of analysis with a t-test for independent samples, which may suggest a tendency for eyewitnesses to remember the male perpetrator poorer than the observers. These results are consistent with recognition memory rates, suggesting that under the influence of negative emotions, memory of the appearance of the violent perpetrator may be impaired.

Table C1

Detailed analysis of FRM relating to perpetrators look presented by gender of the perpetrator

	M (*SD*)				Significance	
'Perpetrator'	Crime	Neutral	*t*	*df*	One-tailed *p*	Two-tailed *p*
Male	3.11 (2.11)	3.73 (2.50)	-1.66	148	.050	.99
Female	1.77 (1.53)	1.91 (1.59)	-.52	148	.301	.601

Additional Analysis D: Other measures of emotionality

In addition to utilizing the Emotionality Style Questionnaire (ESQ) to assess emotionality, participants also completed the HEXACO-60 questionnaire (Ashton & Lee, 2009), offering a comprehensive evaluation of personality traits. In our exploratory analysis, we specifically examined the Emotionality domain, with a focus on facet-level factors: Anxiety and Fearfulness. Table D1 in the supplement showcases the correlations between ESQ and HEXACO measures. The significance of these correlations suggests a partial overlap between the constructs assessed by the two measures, prompting further investigation.

To delve deeper into these variables, we conducted moderation analyses with Anxiety and Fearfulness as moderators influencing the relationship between condition and memory measures. However, none of the interactions between these variables were deemed significant, as shown in Table D2. The only variable trending toward significance is Anxiety, particularly in the model investigating the number of details about the interaction between Perpetrators and Victims as the outcome variable

Table D1

Correlation coefficients between the Emotionality Style Questionnaire (ESQ) and HEXACO factors and domain

		HEXACO Anxiety	HEXACO Fearfulness	HEXACO Emotionality	Healthy Emotionality
HEXACO Anxiety	Pearson r	1	.441**	.728**	-.366**
	Sig.		<.001	<.001	<.001
	N	150	150	150	150
HEXACO	Pearson r	.441**	1	.746**	-.209*
	Sig.	<.001		<.001	.010

Fearfulness	N	150	150	150	150
HEXACO Emotionality domain	Pearson r	.728**	.746**	1	-.253**
	Sig.	<.001	<.001		.002
	N	150	150	150	150
ESQ Healthy Emotionality	Pearson r	-.366**	-.209*	-.253**	1
	Sig.	<.001	.010	.002	
	N	150	150	150	150

**. Correlation is significant at the 0.01 level (2-tailed).

*. Correlation is significant at the 0.05 level (2-tailed).

Table D2

A summary of the additional moderation analysis (N=150) performed on standardized variables.

Moderator * X		FRM		
		General	'Perpetrators' look and behavior	Interaction with 'Victim'
Emotionality (HEXACO)	*β*	.054	.025	.103
	se	.082	.080	.073
	t	.655	.313	1.401
	p	.513	.754	.163
	95% Cl	-.108; .216	-.108; .216	-.042; .248
Fearfulness (HEXACO)	*β*	.001	-.059	.062
	se	.083	.081	.075
	t	.012	-.724	.833
	p	.990	.470	.406
	95% Cl	-.164; .166	-.218; .101	-.086; .210
	β	.063	.048	.126

Anxiety (HEXACO)	se	.081	.080	.072
	t	.771	.609	1.743
	p	.442	.543	.083
	95% Cl	-.098; .224	-.109; .206	-.017; .269

Additional Analysis E: Gender effects in Face identification

In addition to a basic analysis of face identification, we also checked the identification results with regard to the sex of a subject. We wanted to see if the effect of own-sex/own-sex bias was discernible in our sample. The table below shows the results of the identification by gender of the subjects.

		Condition			
		Criminal		Neutral	
		Sex			
		F	M	F	M
Female face	miss	19	12	23	11
	hit	29	15	25	16
	%	60%	56%	52%	59%
Male face	miss	33	16	21	13
	Hit	15	11	27	14
	%	31%	41%	56%	52%

Note: % indicates percent of correctly identified perpetrator.

Based only on the percentage of correct identification, it can be seen that, for a neutral scene, participants tended to prefer faces of the opposite sex. On the other hand, in the Criminal condition, it seems that they generally misidentified the male perpetrator.

The chi-square test conducted by gender, presented in the table below, in turn, indicates that the general Face identification scores discussed in the main body of this research are responsible for the results obtained among women.

	Perpetrator	
	F	M

Subjects	F	χ2 (1, 96) = .677; p = .411	**χ2 (1, 96) = 6.095; p = .014**
	M	χ2 (1, 54) = .076; p = .7883	χ2 (1, 54) = .670; p = .413

References

Ashton, M. C., & Lee, K. (2009). The HEXACO–60: A short measure of the major dimensions of personality. *Journal of personality assessment*, *91*(4), 340-345.

Benjamini, Y., & Hochberg, Y. (1995). Controlling the False Discovery Rate: A Practical and Powerful Approach to Multiple Testing. *Journal of the Royal Statistical Society. Series B (Methodological)*, *57*(1), 289–300. https://www.jstor.org/stable/2346101

Braithwaite, J. J., Watson, D. G., Jones, R., & Rowe, M. (2013). A Guide for Analysing Electrodermal Activity (EDA) & Skin Conductance Responses (SCRs) for Psychological Experiments. *Psychophysiology*, *49*(1), 1017–1034.

Herlitz, A., & Lovén, J. (2013). Sex differences and the own-gender bias in face recognition: A meta-analytic review. *Visual Cognition*, *21*(9–10), 1306–1336. https://doi.org/10.1080/13506285.2013.823140

Hossein Aqajari, S. A., Naeini, E. K., Mehrabadi, M. A., Labbaf, S., Dutt, N., & Rahmani, A. M. (2021). pyEDA: An Open-Source Python Toolkit for Pre-processing and Feature Extraction of Electrodermal Activity. *Procedia Computer Science*, *184*, 99–106. https://doi.org/10.1016/j.procs.2021.03.021

Mason, S. E. (1986). Age and gender as factors in facial recognition and identification. *Experimental Aging Research*, *12*(3), 151–154. https://doi.org/10.1080/03610738608259453

Sacharin, V., Schlegel, K., & Scherer, K. R. (2012). *Geneva Emotion Wheel Rating Study*. https://archive-ouverte.unige.ch/unige:97849
